# Supplementary material for: Allosteric regulation and crystallographic fragment screening of SARS-CoV-2 NSP15 endoribonuclease
Source: Nucleic Acids Res. 2023 Apr 28;51(10):5255–70. doi: 10.1093/nar/gkad314 (PMC10250223; doi:10.1093/nar/gkad314)
Supplement: gkad314_Supplemental_Files [file gkad314_supplemental_files.zip › Supplementary datasets.zip!/139/Refine_1/01-BUSTER/Cycle-5/shell.01/R_Rfree.html]

# Rxpct factors in resolution bins at cycle 0


|  |  |  |  |  |  |  |  |  |
| --- | --- | --- | --- | --- | --- | --- | --- | --- |
|  | **<d> (Å)**  Rxpct\_Work | Rxpct\_Free | Rxpct\_All | |  |  |  |  |
|  | 13.2733 | 0.3172 | 0.4774 | 0.3240 |  |
|  | 7.6634 | 0.2575 | 0.2730 | 0.2584 |  |
|  | 5.9360 | 0.2723 | 0.2865 | 0.2729 |  |
|  | 5.0168 | 0.2130 | 0.2220 | 0.2135 |  |
|  | 4.4244 | 0.1856 | 0.2183 | 0.1873 |  |
|  | 4.0021 | 0.1813 | 0.1808 | 0.1813 |  |
|  | 3.6814 | 0.1879 | 0.2321 | 0.1900 |  |
|  | 3.4272 | 0.1884 | 0.2039 | 0.1892 |  |
|  | 3.2193 | 0.1812 | 0.2174 | 0.1832 |  |
|  | 3.0451 | 0.1810 | 0.2070 | 0.1822 |  |
|  | 2.8965 | 0.1850 | 0.2145 | 0.1863 |  |
|  | 2.7677 | 0.1837 | 0.2071 | 0.1848 |  |
|  | 2.6547 | 0.1916 | 0.2184 | 0.1932 |  |
|  | 2.5545 | 0.1911 | 0.2123 | 0.1923 |  |
|  | 2.4648 | 0.1887 | 0.2130 | 0.1898 |  |
|  | 2.3840 | 0.1949 | 0.2292 | 0.1965 |  |
|  | 2.3106 | 0.1931 | 0.1844 | 0.1927 |  |
|  | 2.2436 | 0.1968 | 0.2113 | 0.1976 |  |
|  | 2.1821 | 0.1917 | 0.1889 | 0.1915 |  |
|  | 2.1254 | 0.2017 | 0.2019 | 0.2017 |  |
|  | **Overall** | 0.2035 | 0.2286 | 0.2048 |  |

# Rxpct factors in resolution bins at cycle 1


|  |  |  |  |  |  |  |  |  |
| --- | --- | --- | --- | --- | --- | --- | --- | --- |
|  | **<d> (Å)**  Rxpct\_Work | Rxpct\_Free | Rxpct\_All | |  |  |  |  |
|  | 13.2733 | 0.3174 | 0.4774 | 0.3242 |  |
|  | 7.6634 | 0.2577 | 0.2718 | 0.2585 |  |
|  | 5.9360 | 0.2725 | 0.2857 | 0.2731 |  |
|  | 5.0168 | 0.2129 | 0.2230 | 0.2135 |  |
|  | 4.4244 | 0.1856 | 0.2187 | 0.1874 |  |
|  | 4.0021 | 0.1813 | 0.1806 | 0.1812 |  |
|  | 3.6814 | 0.1878 | 0.2322 | 0.1899 |  |
|  | 3.4272 | 0.1883 | 0.2040 | 0.1891 |  |
|  | 3.2193 | 0.1811 | 0.2174 | 0.1831 |  |
|  | 3.0451 | 0.1808 | 0.2069 | 0.1821 |  |
|  | 2.8965 | 0.1848 | 0.2145 | 0.1862 |  |
|  | 2.7677 | 0.1835 | 0.2071 | 0.1846 |  |
|  | 2.6547 | 0.1914 | 0.2182 | 0.1931 |  |
|  | 2.5545 | 0.1910 | 0.2122 | 0.1922 |  |
|  | 2.4648 | 0.1886 | 0.2130 | 0.1897 |  |
|  | 2.3840 | 0.1949 | 0.2293 | 0.1965 |  |
|  | 2.3106 | 0.1931 | 0.1846 | 0.1927 |  |
|  | 2.2436 | 0.1968 | 0.2113 | 0.1976 |  |
|  | 2.1821 | 0.1917 | 0.1891 | 0.1916 |  |
|  | 2.1254 | 0.2017 | 0.2020 | 0.2017 |  |
|  | **Overall** | 0.2035 | 0.2286 | 0.2048 |  |

# Rxpct factors in resolution bins at cycle 2


|  |  |  |  |  |  |  |  |  |
| --- | --- | --- | --- | --- | --- | --- | --- | --- |
|  | **<d> (Å)**  Rxpct\_Work | Rxpct\_Free | Rxpct\_All | |  |  |  |  |
|  | 13.2733 | 0.3172 | 0.4771 | 0.3240 |  |
|  | 7.6634 | 0.2573 | 0.2714 | 0.2582 |  |
|  | 5.9360 | 0.2721 | 0.2853 | 0.2726 |  |
|  | 5.0168 | 0.2126 | 0.2227 | 0.2132 |  |
|  | 4.4244 | 0.1855 | 0.2186 | 0.1872 |  |
|  | 4.0021 | 0.1812 | 0.1806 | 0.1811 |  |
|  | 3.6814 | 0.1877 | 0.2322 | 0.1898 |  |
|  | 3.4272 | 0.1881 | 0.2041 | 0.1890 |  |
|  | 3.2193 | 0.1809 | 0.2173 | 0.1829 |  |
|  | 3.0451 | 0.1806 | 0.2067 | 0.1818 |  |
|  | 2.8965 | 0.1846 | 0.2145 | 0.1860 |  |
|  | 2.7677 | 0.1833 | 0.2070 | 0.1844 |  |
|  | 2.6547 | 0.1912 | 0.2181 | 0.1928 |  |
|  | 2.5545 | 0.1909 | 0.2121 | 0.1921 |  |
|  | 2.4648 | 0.1884 | 0.2130 | 0.1896 |  |
|  | 2.3840 | 0.1948 | 0.2293 | 0.1964 |  |
|  | 2.3106 | 0.1931 | 0.1846 | 0.1927 |  |
|  | 2.2436 | 0.1968 | 0.2112 | 0.1975 |  |
|  | 2.1821 | 0.1917 | 0.1891 | 0.1916 |  |
|  | 2.1254 | 0.2018 | 0.2019 | 0.2018 |  |
|  | **Overall** | 0.2034 | 0.2285 | 0.2046 |  |

# Rxpct factors in resolution bins at cycle 3


|  |  |  |  |  |  |  |  |  |
| --- | --- | --- | --- | --- | --- | --- | --- | --- |
|  | **<d> (Å)**  Rxpct\_Work | Rxpct\_Free | Rxpct\_All | |  |  |  |  |
|  | 13.2733 | 0.3129 | 0.4781 | 0.3199 |  |
|  | 7.6634 | 0.2525 | 0.2600 | 0.2530 |  |
|  | 5.9360 | 0.2642 | 0.2934 | 0.2654 |  |
|  | 5.0168 | 0.2082 | 0.2163 | 0.2087 |  |
|  | 4.4244 | 0.1826 | 0.2145 | 0.1843 |  |
|  | 4.0021 | 0.1792 | 0.1802 | 0.1793 |  |
|  | 3.6814 | 0.1856 | 0.2277 | 0.1876 |  |
|  | 3.4272 | 0.1863 | 0.2055 | 0.1873 |  |
|  | 3.2193 | 0.1784 | 0.2159 | 0.1804 |  |
|  | 3.0451 | 0.1780 | 0.2062 | 0.1793 |  |
|  | 2.8965 | 0.1825 | 0.2159 | 0.1841 |  |
|  | 2.7677 | 0.1814 | 0.2076 | 0.1826 |  |
|  | 2.6547 | 0.1887 | 0.2168 | 0.1904 |  |
|  | 2.5545 | 0.1894 | 0.2079 | 0.1905 |  |
|  | 2.4648 | 0.1868 | 0.2087 | 0.1878 |  |
|  | 2.3840 | 0.1943 | 0.2257 | 0.1958 |  |
|  | 2.3106 | 0.1933 | 0.1850 | 0.1929 |  |
|  | 2.2436 | 0.1975 | 0.2126 | 0.1983 |  |
|  | 2.1821 | 0.1926 | 0.1882 | 0.1924 |  |
|  | 2.1254 | 0.2030 | 0.2014 | 0.2029 |  |
|  | **Overall** | 0.2007 | 0.2264 | 0.2020 |  |
